# Comparative Effectiveness of Radiotherapy versus Focal Laser Ablation in Patients with Low and Intermediate Risk Localized Prostate Cancer

**DOI:** 10.1038/s41598-020-65863-8

**Published:** 2020-06-04

**Authors:** Xianghong Zhou, Kun Jin, Shi Qiu, Di Jin, Xinyang Liao, Xiang Tu, Xiaonan Zheng, Jiakun Li, Lu Yang, Qiang Wei

**Affiliations:** Department of Urology, Institute of Urology, National Clinical Research Center for Geriatrics and Center of Biomedical big data, West China Hospital of Sichuan University, Chengdu, Sichuan Province China

**Keywords:** Prostate, Cancer

## Abstract

At present, focal laser ablation (FLA) as a new PCa local treatment has attracted attention. We aim at comparing the survival outcomes between radiotherapy (RT) and FLA to reveal whether FLA can be used as an alternative to RT for patients with low and intermediate-risk localized PCa.We conducted analyses with data from the SEER database (2004–2015). Propensity score matching and instrumental variate (IV) were used to reduce the influence of bias and unmeasured confounders maximally.In the adjusted multivariate regression, FLA had lower overall survival (OS) benefits (HR = 1.49; 95%CI: 1.18–1.87; p < 0.001). After propensity score matching, RT still had better OS (HR = 1.50; 95%CI: 1.17–1.93; p = 0.001). The outcomes of IV-adjusted analysis showed FLA was significantly inferior to RT in OS (HR = 1.49; 95%CI: 1.18–1.87). In the subgroup analyses, for those with PSA < 4 ng/mL, FLA showed markedly worse OS and cancer-specific mortality (CSM) outcomes (OS HR = 1.89; 95%CI: 1.01–3.53; p = 0.0466 and CSM HR = 4.25; 95%CI: 1.04–17.43; p = 0.044).FLA is a promising focal therapy of PCa. But our research demonstrated RT still had an obvious advantage in survival benefits over FLA. Using FLA as an alternative treatment for RT requires careful consideration by clinicians.

## Introduction

Prostate cancer (PCa) is the second most frequent cancer among males, which caused roughly 358,989 deaths in 2018^1^. Meanwhile, the popularization of prostate specific antigen (PSA) screening has increased the diagnosis of low-risk and intermediate-risk localized PCa globally^2^. According to the statistics from the United States in 2014, patients diagnosed as low and intermediate risk PCa accounted for 74.0% of all PCa patients^3^. Therefore, reasonable treatments of such patients are currently very important.

Other than Active surveillance (AS), Radiotherapy (RT) and Radical prostatectomy (RP) are the standard active treatments for patients with low-risk or intermediate-risk localized prostate cancer recommended by current clinical guidelines^4^. However, several important follow-up studies found that whether the patients were treated with RP or RT, many patients had problems with urinary incontinence, erectile dysfunction, or intestinal complications throughout early, intermediate, and long-term follow-up^5,6^.

In order to improve the quality of life of patients, a new focal therapy, focal laser ablation (FLA), has been developed to ablate tumors selectively while sparing the neurovascular bundles, sphincter, and urethra for better functional outcomes^7^. A recent phase II clinical trial reported that FLA was associated with favorable short-term oncologic outcomes with no major urinary, sexual, or bowel side effects^8^. Another small-scale longitudinal outcome study for patients with localized PCa also showed that FLA could achieve early oncologic control of localized PCa with few complications or adverse effects on quality of life^9^. A larger retrospective study involving 120 patients reported that the 1-year retreatment free rate of patients who had received FLA was 83%, and the sexual and urinary function did not significantly change after FLA^10^.

Although the short-term oncologic and functional outcomes of FLA are encouraging, current trials of FLA for PCa are all short-term single-arm studies without long-term oncologic outcomes, overall survival (OS) data or cancer-specific mortality (CSM) data. And the number of patients included in the trials is also small. Whether FLA can bring long-term survival benefits equivalent to conventional treatments like RT for patients is still unclear.

To circumvent these defects, we evaluated the overall survival and prostate cancer-specific mortality at long-term follow-up in the comparison of patients treated with FLA versus patients treated with RT.

## Results

### Patient characteristics

**Figure 1:**
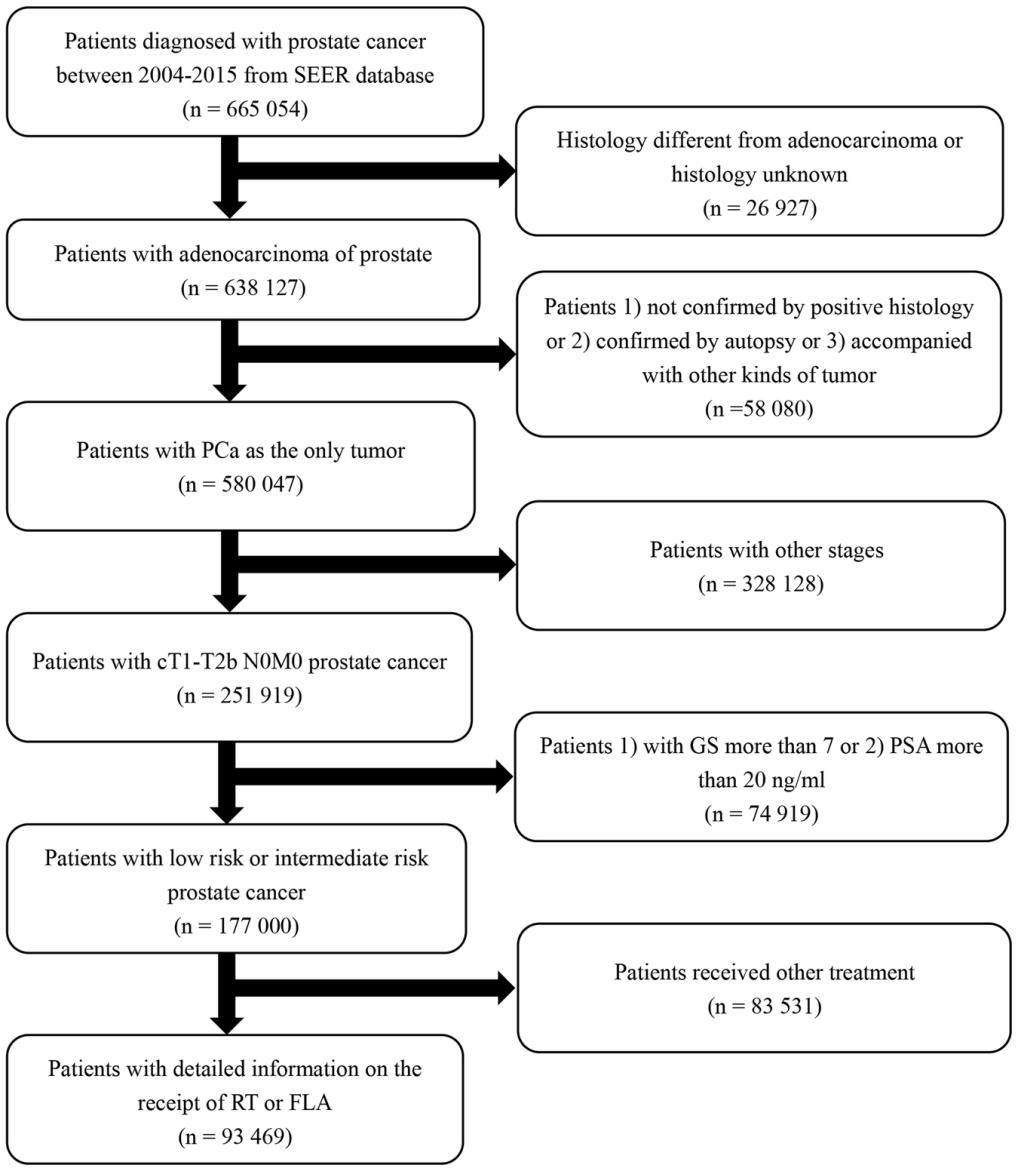
Flowchart describing the selection of patients in the Surveillance, Epidemiology, and End Results database, 2004–2015.

A total of 93,469 patients were included in the analysis, including 93,041 patients treated with radiation therapy and 428 patients treated with laser ablation. Patients’ characteristics were shown in Table 1. Comparing patients who were treated with laser ablation versus those undergone radiotherapy, patients treated with laser ablation were more likely to have older age (p < 0.001), lower PSA (p < 0.05), and lower GS (p < 0.05). There were no differences between both groups on T stage (p = 0.277) and race (p = 0.754). In the radiation therapy cohort, 81,015 patients died, and 1,303 of whom died of prostate cancer-specific reasons. In the focal laser ablation cohort, 356 patients died, and 7 of whom died of prostate cancer-specific reasons. Inclusion and exclusion criteria were shown in the flowchart in detail (Fig. 1).

**Table 1:** Descriptive characteristics of 93,469 patients undergone radiotherapy or focal laser ablation between 2004 and 2015 from the Surveillance Epidemiology and End Results database.

	RT (N = 93041)	FLA (N = 428)	p value
**Age, yr mean** ± **SD**	66.66 ± 7.73	70.07 ± 8.56	<0.001
**PSA level (ng/ml), mean** ± **SD**	7.05 ± 3.41	6.71 ± 3.62	0.045
**Marital status, n (%)**			0.005
Married	64723 (69.56%)	278 (64.95%)	
Single	9793 (10.53%)	50 (11.68%)	
Divorced/Widowed	10861 (11.67%)	72 (16.82%)	
Unknown	7664 (8.24%)	28 (6.54%)	
**Race, n (%)**			0.754
White	70481 (75.75%)	328 (76.64%)	
Black	16764 (18.02%)	78 (18.22%)	
Other	4537 (4.88%)	16 (3.74%)	
Unknown	1259 (1.35%)	6 (1.40%)	
**T stage, n (%)**			0.277
T1	82281 (88.44%)	389 (90.89%)	
T2a	7784 (8.37%)	29 (6.78%)	
T2b	2976 (3.20%)	10 (2.34%)	
**Gleason Score, n (%)**			0.045
3 + 3	50100 (53.85%)	255 (59.58%)	
3 + 4	29796 (32.02%)	125 (29.21%)	
4 + 3	13145 (14.13%)	48 (11.21%)	

Abbreviations: SD = standard difference, PSA = prostate-specific antigen, RT = radiotherapy, FLA = focal laser ablation.

### Survival outcomes according to different treatments

**Figure 2:**
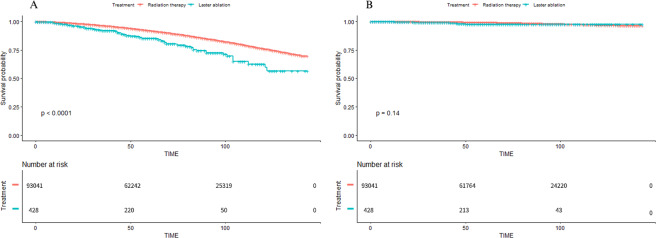
Kaplan-Meier survival curves of primary cohorts (RT VS FLA). A, Kaplan-Meier survival curve of OS in the comparison of RT and FLA. B, Kaplan-Meier survival curve of CSS in the comparison of RT and FLA. Abbreviation: OS = Overall survival, CSS = Cancer-specific survival, RT = Radiation therapy, FLA = Focal laser ablation. Abbreviation: RT = radiotherapy, FLA = focal laser ablation.

From the multivariate regression analysis, FLA was associated with worse OS benefits compared with RT (Hazard ratio [HR] = 1.91; 95% confidence interval [CI]: 1.51–2.40; p < 0.001). After adjusting for the covariates including age, T stage, PSA level, GS, the results did not change significantly (HR = 1.49; 95%CI: 1.18–1.87; p < 0.001). These two treatments were evaluated in multivariate regression analysis of CSM as well. The results showed that there was no significant difference in the extent of which FLA and RT reduced CSM (HR = 1.73; 95%CI: 0.82–3.64; p = 0.147). After adjusting confounding factors, FLA and RT still maintained the same effects in reducing CSM (HR: 1.57; 95% Cl: 0.74–3.29; p = 0.237). According to the Kaplan-Meier curves, same as previous analyses, FLA performed significantly worse than RT in increasing OS, while there was no obvious difference in reducing CSM compared with RT (Fig. 2).

### Survival analysis after propensity score matching

**Figure 3:**
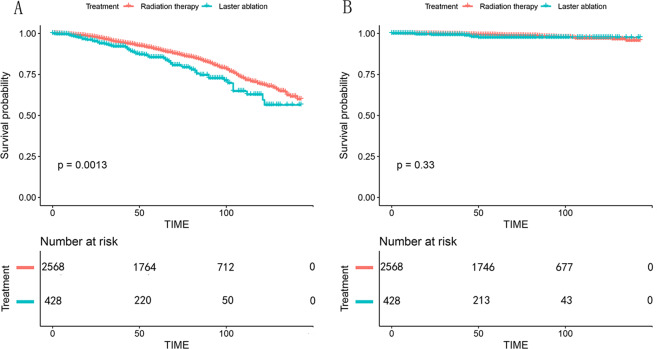
Kaplan-Meier survival curves of matched cohorts (RT VS FLA). A, Kaplan-Meier survival curve of OS in the comparison of RT and FLA. B, Kaplan-Meier survival curve of CSS in the comparison of RT and FLA. Abbreviation: OS = Overall survival, CSS = Cancer-specific survival, RT = Radiation therapy, FLA = Focal laser ablation. Abbreviation: RT = radiotherapy, FLA = focal laser ablation.

Following the propensity score matching (PSM), 2,568 RT-treated patients matched 428 FLA-treated patients. (Supplementary Table 1) The statistical differences in baseline characteristics between the two groups were eliminated after PSM (Table 2). In the matched radiation therapy cohort, 2,133 patients died, and 41 of whom died of prostate cancer-specific reasons. In the focal laser ablation cohort, 356 patients died, and 7 of whom died of prostate cancer-specific reasons. We performed another multivariate regression analysis in the matched cohort for OS and CSM, patients treated with RT still had better overall survival benefits (HR = 1.50; 95%CI: 1.17–1.93; p = 0.001), but RT and FLA remained consistent in reducing CSM performance with no significant difference (HR = 1.48; 95%CI: 0.66–3.32; p = 0.336). Kaplan-Meier analysis was performed in the matched cohort, and the results also showed that RT was better than FLA in OS, but there was no significant difference in CSS (Fig. 3).

**Table 2:** Descriptive characteristics of 2,568 patients received radiotherapy versus 428 patients received focal laser ablation after propensity score matching (ratio 4:1) between 2004 and 2015 from the Surveillance Epidemiology and End Results database.

	RT (N = 2568)	FLA (N = 428)	p value
**Age, yr mean** ± **SD**	69.89 ± 8.38	70.07 ± 8.56	0.677
**PSA level (ng/ml), mean** ± **SD**	6.62 ± 3.30	6.71 ± 3.62	0.603
**Marital status**			0.009
Married	1803 (70.2)	278 (65)	
Single	260 (10.1)	50 (11.7)	
Divorced/Widowed	297 (11.6)	72 (16.8)	
Unknown	208 (8.1)	28 (6.5)	
**Race**			0.252
White	2007 (78.2)	328 (76.6)	
Black	389 (15.1)	78 (18.2)	
Other	136 (5.3)	16 (3.7)	
Unknown	36 (1.4)	6 (1.4)	
**T stage**			0.822
T1	2311 (90)	389 (90.9)	
T2a	186 (7.2)	29 (6.8)	
T2b	71 (2.8)	10 (2.3)	
**GS biopsy**			0.613
3 + 3	1582 (61.6)	255 (59.6)	
3 + 4	733 (28.5)	125 (29.2)	
4 + 3	253 (9.9)	48 (11.2)	
**Low risk vs intermediate risk**			0.184
Low risk	1430 (55.7)	223 (52.1)	
Intermediate risk	1138 (44.3)	205 (47.9)	

Abbreviations: SD = standard difference, PSA = prostate-specific antigen, RT = radiotherapy, FLA = focal laser ablation.

### Survival outcomes from further statistical analysis

The outcomes of instrument variate (IVA) adjusted analysis showed that the ability of FLA to improve OS was still significantly inferior to RT (HR = 1.49; 95%CI: 1.18–1.87). And same as previous analysis, there was no obvious difference of CSM in the comparison of RT and FLA (HR = 1.57; 95%CI: 0.74–3.29) (Table 3).

**Table 3:** Multivariate cox regression analyses for OS and CSM in the total cohort and matched population.

Outcome	Treatment	Non-adjusted model	Adjusted model	PSM model	IVA-adjusted model
OS	RT	Ref.	Ref.	Ref.	Ref.
	FLA	1.91 (1.51, 2.40) p < 0.001	1.49 (1.18, 1.87) p < 0.001	1.50 (1.17, 1.93) p = 0.001	1.49 (1.18, 1.87)
**CSM**	**RT**	**Ref**.	**Ref**.	**Ref**.	**Ref**.
	FLA	1.73 (0.82, 3.64) p = 0.147	1.57 (0.74, 3.29) p = 0.237	1.48 (0.66, 3.32) p = 0.336	1.57 (0.74, 3.29)

Abbreviations:

OS = overall survival, CSM = cancer specific mortality, RT = radiotherapy, FLA = focal laser ablation, PSM = propensity score matching, IVA = instrument variable

Adjusted model: age, T stage, Gleason score (GS) and prostate specific antigen (PSA) level

Propensity score matching (PSM) model: matched according to age, T stage, GS and PSA

Instrument variate (IVA) adjusted model: adjusted for age, T stage, GS and PSA and residual.

**Figure 4:**
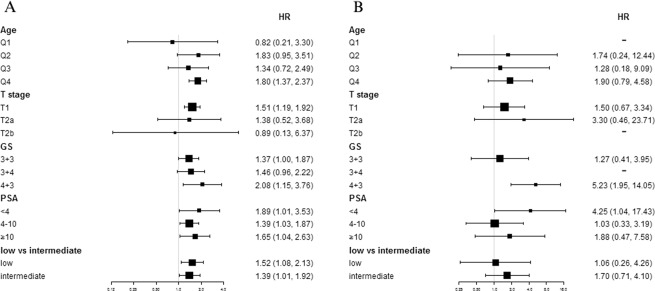
Subgroup analyses of OM and CSM (RT VS FLA). A, Subgroup analysis of OM in the comparison of RT and FLA. B, Subgroup analysis of CSM in the comparison of RT and FLA. Abbreviation: OM = Overall Mortality, CSM = Cancer Specific Mortality, RT = Radiotherapy, FLA = Focal laser ablation, GS = Gleason Score, PSA = Prostate Specific Antigen, Q1-Q4 = Quartile 1 - Quartile 4.

Our subgroup analyses showed patients with different T stage, PSA levels and Gleason scores had a different degree of reactions to FLA and RT. For those with T stage of T1, the OS of FLA was obviously worse than RT, showing that RT can bring significant OS benefits (HR = 1.51; 95%CI: 1.19–1.92; p < 0.001). The CSM outcomes had no significant difference between the two treatments (HR = 1.50; 95%CI: 0.67–3.34; p < 0.001). Similarly, FLA showed markedly worse OS and CSM outcomes compared with RT for those with PSA < 4 ng/mL (OS HR = 1.89; 95%CI: 1.01–3.53; p = 0.047 and CSM HR = 4.25; 95%CI: 1.04–17.43; p = 0.044). Notably, for patients with GS 4 + 3, RT performed significantly better than FLA in both OS and CSM (OS HR = 2.08; 95%CI: 1.15–3.76; p = 0.016 and CSM HR = 5.23; 95%CI: 1.95–14.05; p = 0.001) (Fig. 4).

As for sensitivity analysis, the inverse probability of treatment weighting (IPTW) adjusted model showed that FLA may be both related to a higher risk of overall death and CSM. (OS HR = 1.40; 95%CI: 1.36–1.44; p < 0.001 and CSM HR = 1.21; 95%CI: 1.12–1.31; p < 0.001). Meanwhile, the results of the standardized mortality ratio weighting (SMRW) adjusted model also indicated that RT had advantages over FLA in improving OS of patients (HR = 1.44; 95%CI: 1.03–2.02; p = 0.032) (Supplementary Table 2).

## Discussion

FLA is a new organ-preserving treatment of PCa that aims to reduce complications and improve patients’ quality of life without affecting oncological control. The theory of FLA is to induce coagulation necrosis of tumor cells by heating of targeted tissue, using the high- energy delivered by laser fibers inserted through needles^9,11^.

Our study included long-term survival data from 428 FLA-treated patients and 93041 RT-treated patients and compared OS and CSM outcomes between RT and FLA. Our results showed that patients received RT treatment can achieve significantly better long-term OS than those received FLA. Although these two methods performed not much differently in CSM.

Several studies published in recent years showed that FLA performed well on short-term oncologic outcomes and had the ability to reduce complications that could affect the quality of life of patients^9,12,13^.

A Phase II evaluation of FLA including 27 FLA-treated patients reported a favorable oncologic outcome within 1 year. The targeted biopsy of the ablation zone found persistent cancer in only 1/27 men 3 months after treatment and the systematic biopsy found cancer in 10/27 men 12 months after treatment^8^. A larger cohort study of 120 FLA-treated patients reported a significant reduction in PSA levels after a one-year follow-up (pre-FLA mean PSA 6.05 ng/ml and post-FLA mean PSA 3.25 ng/ml, respectively), with only 17% of patients receiving tumor therapy again because of positive biopsy after MR imaging abnormalities^10^. This demonstrated the important role of FLA in limiting disease progression.

At the same time, the two studies above both used International Prostate Symptom Score (IPSS) and Sexual Health Inventory for Men (SHIM) to measure erectile function and urinary function before and after FLA, and the results showed that there was no significant difference. Compared with the decline in quality of life that often occurred in RT or RP, FLA showed its advantages in functional protection^14^. However, these studies still lacked long-term survival outcomes and comparisons of survival benefits between RT or RP.

Through our research on low risk and intermediate risk PCa patients, we could find that although the short-term oncologic and functional outcomes of FLA were excellent, RT was still significantly better than FLA in long-term survival benefits, especially for patients with T stage T1, PSA < 4 ng/mL, and GS 4 + 3. In Fig. 4, we could see that GS 4 + 3 group was the most obvious group that FLA performed inferior to RT. The latest AUA clinical guidelines also indicated that several studies had demonstrated similar results that the prognosis of Gleason Score = 4 + 3 was significantly worse than Gleason Score = 3 + 4. According to this, the intermediate-risk prostate cancer was divided into intermediate prostate cancer with good prognosis (favorable) and intermediate risk prostate cancer with poor prognosis (unfavorable)^4,15–17^. For such patients, focal therapy might no longer be effective in controlling tumor progression, compared with other whole-gland treatments.

There are some reasons why the FLA is significantly worse than RT on long-term OS benefits in the case of a small difference in CSM. First, as a kind focal therapy, FLA has more potential risk for incomplete tumor tissue clearance than whole gland therapy^18^. Interim results of a study of 98 patients and 138 tumor foci treated with FLA reported 23% rate of cancer residual or recurrent cancer^19^. Similarly, in a 2-year follow-up, there were 17/34 men with positive biopsy at 2 years after FLA^13^. Focal therapy is often susceptible to poor navigation, inadequacy of imaging to delineate tumor boundaries and variable precision of tissue destruction^19^. These risk factors of FLA may not be manifested until a long time, and ultimately reflected in the differences in OS. Second, for patients with low risk and intermediate risk PCa, the risk of cancer-specific death is inherently low^20,21^. Therefore, the number of samples of CSM is relatively small compared to OS (Alive or death from other diseases: 98.4% and cancer-specific deaths: 1.6% relatively). At the same time, FLA had obvious higher HR values in CSM, which also indicated that the low difference in CSM should be caused by a small sample size, not the treatments themselves. The low difference in CSM does not indicate that FLA can give patients the same level of survival benefit as RT.

The main advantages of our research are as follows. First, our research samples were of a large number, having an advantage in quantity compared with the reported studies about FLA. Second, as far as we know, our study was the first one that compared the retrospective data of FLA and RT. Third, current researches focused on short-term oncologic control, while our research mainly focused on long-term survival outcomes. We used a series of statistical analyses to reduce bias and confounding factors, confirming that RT could provide more survival benefits than FLA for patients with low and intermediate risk PCa. Our results could provide a reference for future treatment options. Clinicians should consider that RT is a more effective treatment because of its advantages over FLA in survival benefits.

There are still several limitations to our study. First, although we have used varies statistical methods to reduce the bias, it was essentially a retrospective study with a lower level of evidence than randomized controlled trial (RCT). No RCTs on FLA have been reported yet, thus more high-quality RCTs are still needed to better evaluate the effectiveness and safety of FLA, especially RCTs comparing FLA with RT, RP or AS. In addition, clear clinically relevant objectives such as negative biopsy, toxicity, and optimal follow-up schedules also require more RCTs to define^22^. Until these studies are completed, FLA can only be used as an experimental treatment. Second, due to the database’s limitations, patients’ baseline data were not comprehensive and there may be potential confounding factors. We selected IVA to overcome this problem. Third, functional data such as urinary function and erectile function are also absent. There were several clinical trials on FLA mentioned above suggesting that FLA has little effect on patients’ quality of life, while Prostate Testing for Cancer and Treatment (ProtecT) trial reported the negative effects of radiotherapy in erectile dysfunction, decreased bowel function, and urinary voiding and nocturia^23^.

In our study, we selected patients diagnosed during 2004 to 2015. The main reason for this is that the Surveillance, Epidemiology, and End Results (SEER) database began collecting TNM staging information of American Joint Committee on Cancer [AJCC] Cancer Staging Manual in 2004^24^. Using this widely used staging system, we could enable us to more accurately include patients. However, due to the limitation of the time period, we could not ensure that all patients in the RT cohort had received the most modern radiotherapy technology currently available, such as image guided intensity modulated-dose escalated radiation therapy (IMRT). IMRT is the current mainstream radiotherapy technology, the usage of IMRT has increased from 3.1% in 2001 to 64.7% in 2013 in North America^25^. Compared with other radiotherapy technologies like three-dimensional conformal radiotherapy (3D-CRT), some clinical trials have revealed that IMRT has advantages in reducing side effects and biochemical recurrence rate^26,27^. Our study, which included some patients with outdated radiotherapy techniques, still yielded results that RT currently performed better than FLA in survival outcomes. Therefore, we think that the differences in radiotherapy technologies will not significantly affect our findings, and the advantages of RT over FLA may be more obvious in a radiation therapy cohort that includes IMRT only.

## Conclusion

Although several researches have confirmed the excellent performance of FLA in controlling the progression of PCa and functional protection in the short term, for patients with low risk and intermediate risk PCa, RT still provided better long-term survival benefits. In the future, if FLA can solve its current technical shortcomings such as navigation, imaging, and precision, its therapeutic effect may be better with favorable survival benefits and functional protection at the same time. But at present, RT should have a priority over FLA in the management of low risk and intermediate risk PCa.

## Materials and Methods

### Patient selection

From the SEER database, patients with a diagnosis of adenocarcinoma of the prostate (International Classification of diseases-O-3 code: C61.9) between 2004 and 2015 were selected. The TNM stages were assessed by the 7th edition of AJCC Cancer Staging Manual^28^. Inclusion and exclusion criteria were available in Fig. 1.

### Outcomes

The primary endpoint was OS. The secondary endpoint was CSM. CSM included all deaths caused by prostate cancer, complications of treatments, or unknown process in patients with active prostate cancer. Follow-up time was defined as the time between the first treatment of RT or FLA and patient death or last follow-up.

### Statistical analysis

Firstly, the baseline characteristics were compared between the two groups (RT and FLA). A 2-tail t-test was used to reveal the difference in continuous variables, presenting the results as mean ± standard deviation. Likewise, a X^2^ test was used to expose the difference in categorical variables, and the results were presented as the frequency with its proportion.

Secondly, between the two treatment groups, multivariate Cox proportional hazard models were performed to evaluate CSM and OS rates before and after adjusting confounders.

To overcome the selection bias, we performed propensity score matching, of which the propensity scores were reckoned by logistic regression, with both two treatments (RT and FLA) as the outcome and age, T stage, PSA level, Gleason score as the pretreatment and prognostic covariates. When P > 0.05, the baseline characteristics of the matched cohort were considered to be balanced. Meanwhile, we used the Kaplan-Meier method to plot the cumulative incidence survival curve of the original cohort.

To further reduce the impact of selection bias and calculate unmeasured confounders on the outcomes, we also used the regional utilization rate as an instrument variate in the two-stage residual inclusion analysis^29,30^. All covariates and residual were included together to establish a new multivariate Cox proportional hazard model to demonstrate more accurate results.

Several different sensitivity analyses were used to verify the robustness of the results. (1) The analyses of OS and CSM after adjusting propensity scores; (2) Among the whole cohort, IPTW and SMRW methods were used to estimate the relationship between treatment types and outcomes. (3) Analyses of OS and CSM in different groups stratified by propensity scores. (4) Analyses of OS and CSM after the adjustment of unbalanced covariates among the matched cohort.

The statistical software packages R (http://www.R-project.org, The R Foundation) and EmpowerStats (http://www.empowerstats.com, X&Y Solutions, Inc., Boston, MA) were used in the above statistical analyses. A p-value <0.05 was considered statistically significant.

## Data Availability

The data that support the findings of this study are available in SEER dataset at https://seer.cancer.gov/. These data were derived from the following resources available in the public domain: SEER Incidence Data, 1975–2016 https://seer.cancer.gov/data/.

## Supplementray information

Supplementray information.
