# Supplementary material for: Comparative Effectiveness of Radiotherapy versus Focal Laser Ablation in Patients with Low and Intermediate Risk Localized Prostate Cancer
Source: Sci Rep. 2020 Jun 4;10:9112. doi: 10.1038/s41598-020-65863-8 (PMC7272634; doi:10.1038/s41598-020-65863-8)
Supplement: Supplementary file 1 — Supplementray information. [file 41598_2020_65863_MOESM1_ESM.docx]

**Comparative Effectiveness of Radiotherapy versus Focal Laser Ablation in Patients with Low and Intermediate Risk Localized Prostate Cancer**

Xianghong Zhou^1*^, Kun Jin^1*^, Shi Qiu^1,2*^, Di Jin^1^, Xinyang Liao^1^, Xiang Tu^1^, Xiaonan Zheng^1^, Jiakun Li^1^, Lu Yang^1#^, Qiang Wei^1#^

* Xianghong Zhou, Kun Jin, Shi Qiu contributed equally to this study

1. Department of Urology, Institute of Urology, National Clinical Research Center for Geriatrics and Center of Biomedical big data, West China Hospital of Sichuan University, Chengdu, Sichuan Province, China

#Correspondence to:

Pro Qiang Wei, Department of Urology, Institute of Urology, West China Hospital of Sichuan University, No. 37, Guoxue Alley, Chengdu, Sichuan, P.R. China; Post Code: 610041; weiqiang163163@163.com, telephone: +86 18980601425

Pro Lu Yang, Department of Urology, Institute of Urology, West China Hospital of Sichuan University, No. 37, Guoxue Alley, Chengdu, Sichuan, P.R. China; Post Code: 610041; wycleflue@163.com, telephone: +86 13541235213

**Keywords:** Prostate neoplasm; Radiotherapy; Focal laser ablation

**Supplementary Table 1: Propensity score parameter list**

| the variables used in calculating the propensity matching | Age, PSA, GS, T stage | |
| --- | --- | --- |
| Propensity scoring algorithm | Logistic regression model | |
| C-statistical | 0.9618 | |
| Matching method | Greedy matching within specified caliper distances | |
| Distance metric | 0.05 | |
| Matching ratio | (Radiotherapy) 4:1 (Focal laser ablation) | |
| Use of replacement | With replacement | |
| Matching sample size | Radiotherapy: 2,568 cases | Total: 2996 cases |
|  | focal laser ablation: 428 cases |  |

**Supplementary Table 2: sensitivity analysis from propensity score matching (PSM)**

Several models were used to verify the robustness of the treatment effects in the comparison of RT and FLA:

1. Inverse probability of treatment weighting (IPTW) logistic regression model
2. Standard mortality ratio weighting (SMRW) logistic regression model
3. Covariate adjustment propensity score (CAPS) model
4. Propensity score (PPS) stratified model

**Inverse probability of treatment weighting (IPTW)**

IPTW  can be used to compare the effectiveness of 2 or more treatments among the same individuals [1]. It relies on the assumption that the distribution of risk factors in patients received treatment is equal to that found in all individuals, thus making treatment choices balanced [2, 3].

**Standard mortality ratio weighting (SMRW)**

SMRW attempts to estimate the standard effect measure that considers the treatment group as the standard population, making the distribution of risk factors in all patients is equal to that found in the treatment group [4].

**Covariate adjustment propensity score (CAPS)**

After performing propensity score matching calculated by covariates, the covariates in the two treatment groups can be expressed as propensity score. Distribution of several covariates differs after matching (Table 2), indicating that adjustment of PS is necessary for validation.

**Propensity score stratified**

After stratified the whole population into five groups according to propensity score, individual with similar conditions were combined (eg. Those with poor conditions or better conditions).

| RT vs FLA | OS | CSM |
| --- | --- | --- |
| IPTW model |  |  |
| Non-adjusted | 1.35 (1.32, 1.39) | 1.16 (1.08, 1.26) |
| Adjusted | 1.40 (1.36, 1.44) | 1.21 (1.12, 1.31) |
| SMRW model |  |  |
| Non-adjusted | 1.58 (1.13, 2.21) | 1.47 (0.50, 4.32) |
| Adjusted | 1.44 (1.03. 2.02) | 1.43 (0.49, 4.21) |
| CAPS model |  |  |
| Non-adjusted | 1.91 (1.51, 2.40) | 1.73 (0.82, 3.64) |
| Covariate PS as continuous | 1.36 (1.08, 1.72) | 1.58 (0.75, 3.34) |
| Covariate PS as categorical  (divided into five groups) | 1.66 (1.32, 2.10) | 1.65 (0.79, 3.48) |
| PPS stratified model |  |  |
| Q1 | 0.68 (0.17, 2.72) | 0.00 (0.00, Inf) |
| Q2 | 1.66 (0.69, 3.99) | 3.09 (0.43, 22.06) |
| Q3 | 1.98 (1.10, 3.59) | 1.50 (0.21, 10.69) |
| Q4 | 1.33 (0.77, 2.29) | 0.00 (0.00, Inf) |
| Q5 | 1.88 (1.38, 2.56) | 2.83 (1.17, 6.84) |

OS = overall survival, CSM = cancer specific mortality, PPS = propensity score

IPTW model and SMRW model: adjust for age, prostate specific antigen, Gleason score, T stage

CAPS model: adjust for propensity score

References

[1] Curtis LH, Hammill BG, Eisenstein EL, Kramer JM, Anstrom KJ. Using inverse probability-weighted estimators in comparative effectiveness analyses with observational databases. Med Care. 2007;45(10 Supl 2):S103-S107.

[2] Robins JM. Marginal structural models. In: 1997 Proceedings of the Section on Bayesian Statistical Science. Alexandria, VA: American Statistical Association, 1998:1–10.

[3] Robins JM, Hernan MA, Brumback B. Marginal structural models and causal inference in epidemiology. Epidemiology 2000;11:550–60.

[4] Sato T, Matsuyama Y. Marginal structural models as a tool for standardization. Epidemiology 2003;14:680–6.
